# A MITE Transposon Insertion Is Associated with Differential Methylation at the Maize Flowering Time QTL *Vgt1*

**DOI:** 10.1534/g3.114.010686

**Published:** 2014-03-07

**Authors:** Sara Castelletti, Roberto Tuberosa, Massimo Pindo, Silvio Salvi

**Affiliations:** * Department of Agricultural Sciences (DipSA), University of Bologna, Bologna; † Research and Innovation Centre, Foundation Edmund Mach, San Michele all’Adige, Trento, Italy

**Keywords:** DNA methylation, gene regulation, conserved noncoding sequences, *Zea mays*

## Abstract

One of the major quantitative trait loci for flowering time in maize, the *Vegetative to generative transition 1* (*Vgt1*) locus, corresponds to an upstream (70 kb) noncoding regulatory element of *ZmRap2.7*, a repressor of flowering. At *Vgt1*, a miniature transposon (MITE) insertion into a conserved noncoding sequence was previously found to be highly associated with early flowering in independent studies. Because cytosine methylation is known to be associated with transposons and to influence gene expression, we aimed to investigate how DNA methylation patterns in wild-type and mutant *Vgt1* correlate with *ZmRap2.7* expression. The methylation state at *Vgt1* was assayed in leaf samples of maize inbred and F_1_ hybrid samples, and at the syntenic region in sorghum. The *Vgt1*-linked conserved noncoding sequence was very scarcely methylated both in maize and sorghum. However, in the early maize *Vgt1* allele, the region immediately flanking the highly methylated MITE insertion was significantly more methylated and showed features of methylation spreading. Allele-specific expression assays revealed that the presence of the MITE and its heavy methylation appear to be linked to altered *ZmRap2.7* transcription. Although not providing proof of causative connection, our results associate transposon-linked differential methylation with allelic state and gene expression at a major flowering time quantitative trait locus in maize.

Methylation of cytosine residues is one of the most extensively studied epigenetic modifications of DNA. It is widespread in many eukaryotic organisms ranging from fungi to higher plants (Feng *et al.* 2010; Jones 2012). To investigate the role of genic and intergenic methylation, high-resolution DNA methylation analysis has been performed or is underway in many plant species (Zhang *et al.* 2006; Zilberman *et al.* 2007; Cokus *et al.* 2008; Lister *et al.* 2008; Zemach *et al.* 2010; Eichten *et al.* 2011; Li *et al.* 2012; Gent *et al.* 2013). Many of these have shown that transposable elements are highly methylated, which supports the hypothesis that methylation is a strategy to repress TEs mobility (Lippman *et al.* 2003; Zhang *et al.* 2006). Methylation also plays a major role in regulating gene expression and, in general, methylation of promoter sequences has been negatively correlated with transcriptional activity (Zhang *et al.* 2010).

In plants, several studies have highlighted the correlation between epigenetic modifications and heritable phenotypic variation. The presence of 853-bp tandem repeats located 100 kb upstream from the *b1* gene is essential for paramutation (Brink 1958) to occur at the *b1* locus in maize; the methylation state of the repeats correlates with the epigenetic state of the *b1* coding region, which also involves an RNA-mediated mechanism (Stam *et al.* 2002; Arteaga-Vazquez *et al.* 2010). In *Linaria vulgaris*, the methylation state of the *Lcyc* gene was shown to be responsible for a naturally available developmental mutant for flower symmetry (Cubas *et al.* 1999). In melon, the insertion of a DNA transposon in the *CmWIP1* locus, encoding a C2H2 zinc-finger transcription factor, determines hypermethylation of the gene promoter, which in turn results in the formation of female flowers (Martin *et al.* 2009). Telias *et al.* (2011) showed that variegation in apple skin is dependent on differential methylation of the promoter of a myb transcription factor (*MYB10*), which is a key regulator of anthocyanin biosynthesis. On a larger scale, the development of a population of epigenetic recombinant inbred lines in *Arabidopsis thaliana* showed that stably inherited DNA methylation changes are indeed associated with heritable variation for two complex traits, namely flowering time and plant height (Johannes *et al.* 2009).

Flowering time is a key trait for crop adaptation to different cultivation environments and maize is no exception. Genetic dissection of this trait has been the topic of several QTL studies in maize, as the nature of the variability for flowering time is prevalently quantitative (Chardon *et al.* 2004; Buckler *et al.* 2009; Salvi *et al.* 2009; Tuberosa and Salvi 2009). One of the major flowering-time QTL, *Vegetative to generative transition 1* (*Vgt1*) (Phillips *et al.* 1992; Vlăduţu *et al.* 1999; Salvi *et al.* 2002) on chromosome 8, corresponds to a ≈2-kb intergenic region upstream of an *Ap2*-like flowering-time gene, *ZmRap2.7* and appears to act as a *cis*-regulator of *ZmRap2.7* expression (Salvi *et al.* 2007). A maize–sorghum–rice evolutionarily conserved noncoding sequence (CNS) (Freeling and Subramaniam 2009) was identified within *Vgt1*; in early *Vgt1* alleles, this CNS is interrupted by the insertion of a miniature transposon (MITE) belonging to the Tourist family (Salvi *et al.* 2007), and this insertional polymorphism has been repeatedly identified as strongly associated with flowering time in independent studies (Salvi *et al.* 2007; Ducrocq *et al.* 2008; Buckler *et al.* 2009; Hung *et al.* 2012; Truntzler *et al.* 2012).

In this work, we compared the DNA methylation state of two *Vgt1* alleles to investigate a possible role for epigenetic changes at *Vgt1* in the regulation of *ZmRap2.7* expression in the context of a putative *cis*-interaction between the two loci. We focused in particular on the CNS/MITE region with the objective of improving our understanding of the involvement of conserved DNA elements in determining phenotypic variation.

## Materials and Methods

### Plant materials

Seedlings of B73, Gaspé Flint, N28, C22-4, R22, and N28xC22-4 F_1_ hybrid were grown in a greenhouse at 25° under long-day photoperiod. B73 is the reference genotype for the maize community (Wei *et al.* 2007; Schnable *et al.* 2009), N28 is a dent line belonging to the Nebraska Stiff Stalk Synthetic group. The strain C22–4 is nearly isogenic to N28 and carries the early *Vgt1* allele in an ≈7-cM introgression originating from the maize variety Gaspé Flint characterized by extreme earliness (Vlăduţu *et al.* 1999). R22 is an N28 nearly isogenic line derived from the cross between N28 and C22-4 (Salvi *et al.* 2007). For all experiments (methylation analysis and expression analysis), leaf tissue was harvested at four developmental stages: first leaf (V1), third leaf (V3), fifth leaf (V5), and seventh leaf (V7), fully expanded, according to the classification reported in (Ritchie *et al.* 1993); after the collection of leaves, ligule, leaf tip, and midrib vein were removed to keep only leaf blade; tissues from 15 different plants were pooled for each genotype and stage of development. For each genotype and stage of development, two biological replicates were grown and harvested. One additional N28-nearly isogenic line, R66 (as described in Salvi *et al.* 2007) was grown and sampled to be included in methylation analysis (see File S1).

Sorghum B.Tx623 seedlings (seeds kindly provided by William Rooney, Texas A&M University) were grown in a growth chamber in controlled conditions (25°, 16-hr photoperiod). Leaves were harvested from 15 seedlings grown in two reps, at two developmental stages (first leaf, V1 and seventh leaf, V7, fully expanded), and only leaf blades were collected.

### *ZmRap2.7* expression analysis

Total RNA was isolated from leaf tissues by use of TRI Reagent (Sigma-Aldrich, St. Louis, MO). Complementary (c)DNA was synthesized with the High Capacity cDNA Reverse Transcription Kit (Applied Biosystems, Foster City, CA). Real-Time polymerase chain reactions (PCRs) were set up using cDNA as template and Platinum SYBR Green qPCR SuperMix-UDG (Invitrogen, Carlsbad, CA) and performed on a 7500 Fast Real-Time PCR System (Applied Biosystems, Foster City, CA) with the following thermocycling conditions: 40 cycles at 95° for 10’’ and 64° for 1’; primers for the target gene *ZmRap2.7* (Salvi *et al.* 2007) and the housekeeping gene *aat* (alanine aminotransferase, housekeeping gene) (Woodhouse *et al.* 2006) were used (Table S1). Relative gene expression was calculated following the ΔΔCt method (Livak and Schmittgen 2001).

### Bisulfite treatment

Genomic DNA was extracted following a standard CTAB method as described in (Saghai-Maroof *et al.* 1984). For each sample, 500 ng of genomic DNA from N28, C22-4, and N28 x C22-4 F_1_ leaves at V1, V3, V5, and V7 stages and from sorghum B.Tx623 leaves at V1 and V7 stages was treated using EZ DNA Methylation-Gold kit (Zymo Research, Orange, CA) following the manufacturer’s instructions.

### Amplicon ultra-deep sequencing

**Figure 1:**
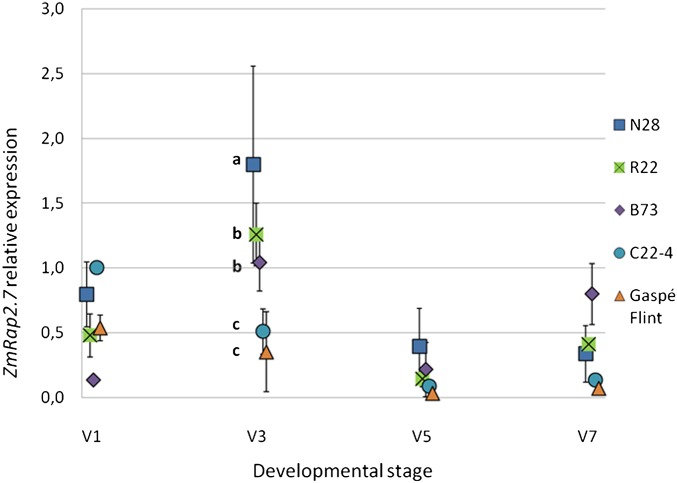
*ZmRap2.7* expression analysis across four developmental stages (V1, V3, V5, and V7) on five maize lines used in this study. Gene expression levels (mean values) are expressed relatively to the *aat* housekeeping gene. Standard deviation values are shown as bars; different letters (a−c) indicate significant difference (*P* < 0.01, Fisher LSD)

Degenerate primers targeting the CNS/MITE region in maize (Figure 1 and Table S1) and the CNS region in sorghum B.Tx623 (Table S1) were designed using the Kismeth software (Gruntman *et al.* 2008). For maize samples, 4 µL of bisulfite-treated genomic DNA was PCR-preamplified with AmpliTaq Gold DNA Polymerase (Applied Biosystems). All the 16 PCR products were quantified by Nanodrop 3300 Fluorospectrometer (Thermo Scientific, Wilmington, DE) using the Quant-iT PicoGreen dsDNA Assay Kit (Invitrogen). The MITE and CNS amplicons were pooled according to the stage of development, and eight different libraries were generated employing the GS-FLX Titanium Rapid Library Preparation Kit following the manufacturer’s recommendations (454 Life Sciences, Branford, CT). To overcome the limitation in the number of samples that can be sequenced in parallel, the Multiplex Identifier was used. For sorghum, after a first preamplification on bisulfite-treated genomic DNA, 1 µL of nonpurified PCR product was used as template in a second nested amplification performed with fusion primers composed of three parts: (i) 454-specific adaptors (A and B), (ii) 10-bp Multiplex Identifier to barcode the samples, and (iii) sequence-specific primers (Table S1); the thermocycling conditions were 30 cycles at 95° for 30”, 49° for 30”, and 72° for 45”. The final double-stranded DNA libraries were quantitated via quantitative (q)PCR using Library quantification kit–Roche 454 Titanium (KAPA Biosystems, Boston, MA) prior to emulsion PCR amplification. Pyrosequencing was performed on a GS FLX instrument (454; Life Sciences) according to the manufacturer’s recommendations. Processed and quality-filtered reads were analyzed with the Kismeth software (Gruntman *et al.* 2008), and statistical significance was tested with analysis of variance. The achieved average depth of sequencing was 1591X for the CNS region in N28, 857X for the CNS/MITE region in C22-4, and 108X for the CNS region in sorghum B.Tx623. Raw data were deposited in the Sequence Read Archive under accession numbers: maize SRX469305 and sorghum SRX469306.

### Methylation quantification with Mutation Surveyor

Two different genomic regions within *Vgt1* (Ampl Bis-Sanger from N28 and C22-4 and CNS/MITE from N28 x C22-4 F_1_, Figure 1 and Table S1) were PCR-amplified, and the final PCR products were run on a 1.5% agarose gel, purified with Wizard SV Gel and PCR Clean-Up System (Promega, Madison, WI) and then sequenced following standard Sanger method. The electropherograms were analyzed with Mutation Surveyor software (Softgenetics, State College, PA) to determine the percentage of methylation for every cytosine residue.

### Allele-specific expression assay

A custom TaqMan SNP Genotyping assay (Applied Biosystems) was designed to discriminate N28 and C22-4 alleles at *ZmRap2.7*. To produce a standard curve, genomic DNAs of N28 and C22-4 were mixed with the following ratios: 4:1, 3:1, 2:1, 1:1, 1:2, 1:3, and 1:4 (N28 allele/C22-4 allele). Following the manufacturer’s protocol, PCRs were performed on these DNA mixes; the log of [FAM intensity (C22-4 allele)/VIC intensity (N28 allele)] was calculated at the last PCR cycle (cycle 40). The standard curve was then generated by linear regression (y = *a* + *b*x), where y is the log of (FAM intensity/VIC intensity) at a given mixing ratio, x is the log of mixing ratio, *a* is the intercept, and *b* is the slope. After RNA extraction from the leaves of N28xC22-4 F_1_ hybrids with the TRI Reagent (Sigma-Aldrich) and cDNA synthesis with the High Capacity cDNA Reverse Transcription Kit (Applied Biosystems), PCRs using the custom TaqMan SNP were performed on cDNA samples; the allele ratio was calculated by intercepting log of (FAM intensity/VIC intensity) on the standard curve (Lo *et al.* 2003).

## Results

### Temporal profile of *ZmRap2.7* expression

Leaf tissues sampled at four different developmental time points (V1, V3, V5, and V7) were used to determine *ZmRap2.7* expression by means of qPCR in five different maize lines: B73, N28, R22 (late flowering, carrying the late *Vgt1* allele) and Gaspé Flint and C22-4 (early flowering, early *Vgt1* allele). The lines B73, N28, and R22, carrying the late *Vgt1* allele, shared a common expression trend, characterized by a peak of *ZmRap2.7* expression at the stage V3, followed by a drop in the levels of *ZmRap2.7* transcripts in ensuing stages (Figure 1). Differently, C22-4 and Gaspé Flint showed maximum *ZmRap2.7* expression at V1. At V3, expression was significantly higher (Fisher’s LSD test *P* < 0.05) in N28 compared with R22 and B73, whose expression levels were in turn significantly greater than in C22-4 and Gaspé Flint.

### Bisulfite sequencing of portions of *Vgt1* in N28 and C22-4 reveals differential methylation and methylation spreading From the MITE transposon

**Figure 2:**
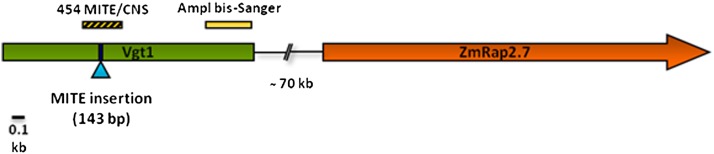
Schematic representation of the *Vgt1-ZmRap2.7* locus (Salvi *et al.* 2007) and of the polymerase chain reaction amplicons used for the bisulfite sequencing DNA methylation analysis. MITE, miniature transposon.

**Figure 3:**
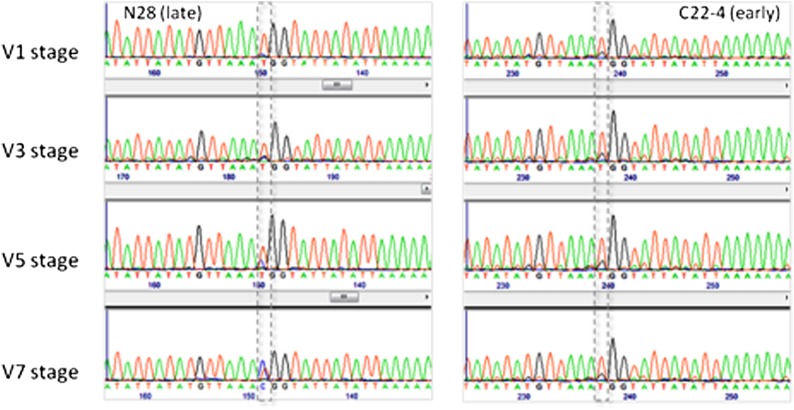
Portion of bisulfite sequencing (Sanger) chromatograms for the ‘Ampl bis-Sanger’ amplicon (1570−1921 bp) showing the differentially methylated cytosine C-1761.

To investigate the dynamics of *Vgt1* DNA methylation levels, we performed a preliminary analysis based on restriction with the methylation-dependent enzyme *Mcr*BC followed by qPCR, which showed a constant in time methylation peak in the central region of *Vgt1*. However, the analysis did not show any methylation difference between the early and late alleles (see File S1, Figure S1, Figure S2, Figure S3, Figure S4, Figure S5, Figure S6). Given the results obtained with this approach, we moved to bisulfite-based methylation analysis. We first focused on the *Vgt1* region encompassing the nucleotides 1570−1921 (Ampl bis-Sanger, see Figure 2). The results showed that the C22-4 (early) *Vgt1* allele is completely unmethylated at all developmental stages, because 54 of the 54 cytosines in the investigated region were converted to thymine. Conversely, for the N28 allele, a single cytosine residue (C-1761) was progressively more methylated during development (10% at stage V1, 16.7% at V3, 30.8% at V5, and 61.5% at V7) (Figure 3).

**Figure 4:**
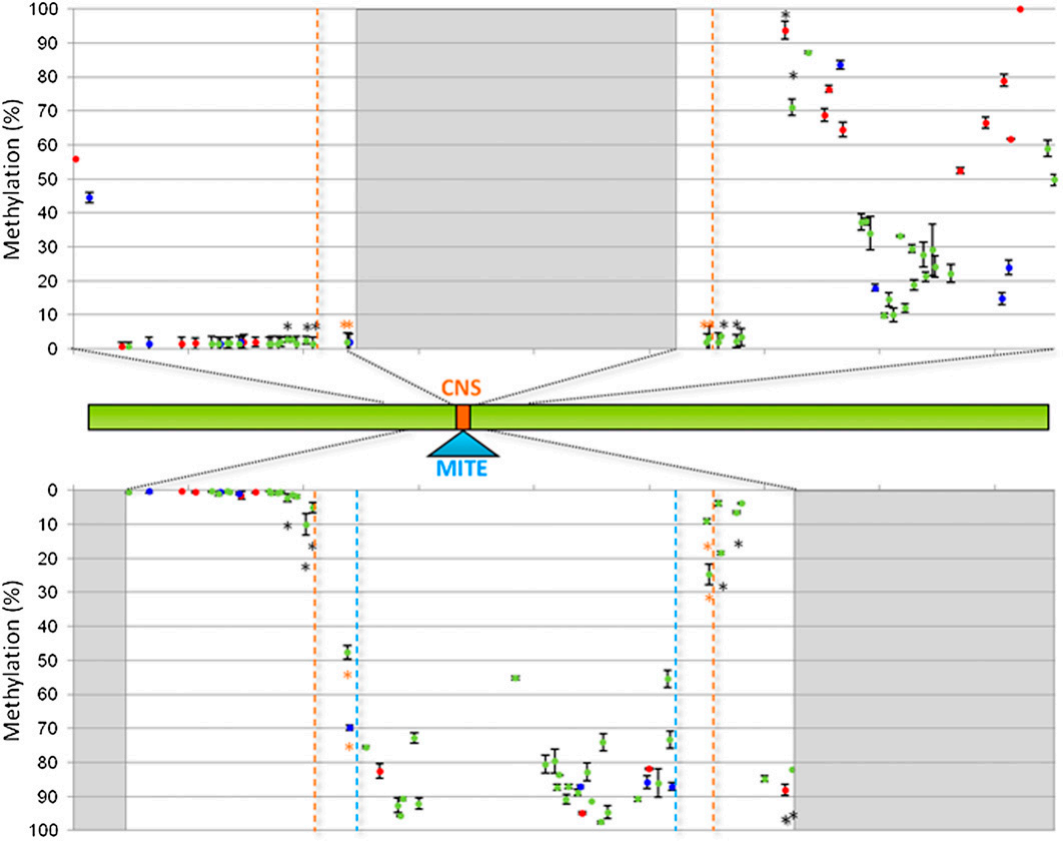
Results of the ultra-deep amplicon bisulfite sequencing at the conserved noncoding sequence/miniature transposon (CNS/MITE) region for the lines N28 and C22-4 at the V3 stage. Mean values are shown; standard deviation values are shown as bars. Methylation data points are represented in different colors, according to cytosine context: red for CG, blue for CHG, green for CHH. Top: methylation level (% of cytosine methylation as estimated by the Kismeth software, black vertical bars) for each cytosine within the sequence 617−920 bp of the N28 (late) allele. The gray block represents the site of MITE insertion (not present in the N28 allele). The orange dotted lines highlight the CNS sequence. Middle: the green bar represents the N28-*Vgt1* locus, with black dotted lines indicating the regions for which methylation has been explored in this experiment. Bottom: methylation level estimated for each cytosine of the C22-4 (early) allele within the region corresponding to the sequence 643−792 bp of the N28 allele. The gray blocks define regions within *Vgt1* that have not been tested in this analysis for the C22-4 allele with respect to N28. The light blue dotted lines delimitate the MITE insertion, which is present in C22-4 only. The black * indicates a significantly differentially methylated cytosine between N28 and C22-4 (*P* < 0.01, LSD). The orange * indicates significant difference in methylation at the cytosine included in the CNS region.

We next investigated a region surrounding the CNS sequence (for the N28 allele, 617−920 bp) or the CNS/MITE insertion (for the C22-4 allele, 292 bp corresponding to the sequence 643−792 bp of the N28 allele plus the MITE) (Figure 3). In both alleles, the region upstream the CNS or CNS/MITE insertion was scarcely methylated; cytosine methylation of the CNS sequence (743−761 bp) was extremely low (1.1%) whereas the MITE itself showed a very high level (85.8%) of average methylation (Average methylation for the three cytosine contexts: 88.8% CG, 89.0% CHG and 85.0% CHH) (Figure 4). N28 and C22-4 showed significant methylation differences among developmental stages; in particular, methylation of the N28 region was higher at V3, compared with earlier and later stages, whereas for C22-4 the most methylated stage corresponded to V7 (*P* < 0.01). The N28 and C22-4 amplicons overlap for 149 nucleotides and among the 31 shared cytosines, 11 showed a significantly different degree of methylation between N28 and C22-4 (*P* < 0.01). Interestingly, the four shared cystosines included in the CNS sequence were differentially methylated between N28 and C22-4 and significantly higher methylation was observed in C22-4 (*P* < 0.01) (Figure 4 and Figure S7).

### Methylation patterns at *Vgt1* are stably maintained

**Figure 5:**
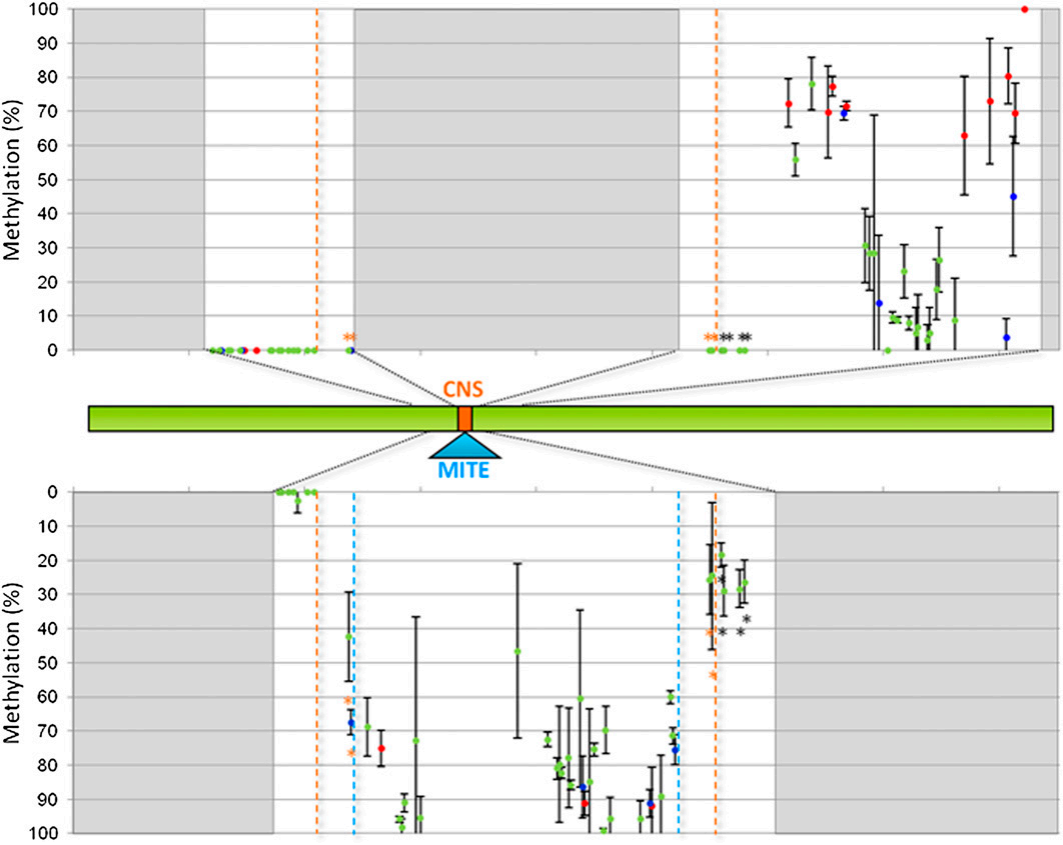
Results of the Sanger bisulfite sequencing at the CNS/MITE region within *Vgt1* for the N28xC22-4 F_1_ hybrid line at the V3 stage. Mean values are shown; standard deviation values are shown as bars. Methylation data points are represented in different colors, according to cytosine context: red for CG, blue for CHG, green for CHH. Top: methylation level (% of cytosine methylation as estimated by the Mutation Surveyor software, black vertical bars) estimated for each cytosine of the N28 (late) allele. Middle: the green bar represents the N28-*Vgt1* locus with black dotted lines indicating the regions for which methylation has been explored in this experiment. Bottom: methylation level estimated for each cytosine of the C22-4 (early) allele. The two light blue dotted lines indicate the MITE insertion; the orange dotted lines highlight the CNS sequence. The black * indicates a significantly differentially methylated cytosine between N28 and C22-4 (*P* < 0.01, two tailed *t*-test). The orange * indicates a significant difference in methylation at the cytosine included in the CNS region. CNS/MITE, conserved noncoding sequence/miniature transposon.

Allele specific methylation analysis of the CNS/MITE region was determined at four developmental stages (V1, V3, V5, and V7) in F_1_ plants obtained by crossing N28 and C22-4. The pattern of methylation of the two allelic forms strongly resembled what was observed in the parental inbred lines: the region upstream the MITE insertion (for the C22-4 allele) or the CNS sequence (in N28) showed weak methylation and the CNS was completely unmethylated (0%), whereas cytosines in all the three possible context were heavily methylated within the MITE transposon (average 84.6% with 85.1% CG, 88.7% CHG, and 84.0% CHH) (Figure 5 and Figure S8). Among the 26 cytosines shared between the two alleles and surveyed in this experiment, eight are more methylated in the C22-4 allele (two tailed *t*-test, *P* < 0.01), this being more evident for the residues adjacent to the MITE transposon. The four cytosines within the CNS displayed a higher level of methylation in C22-4 (*P* < 0.01), as observed in the inbred line-based experiment.

### *ZmRap2.7* allele-specific expression assay

In a previous work, *Vgt1* was shown to be a *cis*-regulator of *ZmRap2.7* transcription (Salvi *et al.* 2007). To test the correlation between cytosine methylation at *Vgt1* and expression of the two alleles of *ZmRap2.7*, we performed an allele-specific TaqMan expression assay on the same N28 × C22-4 F_1_ plants analyzed for methylation. At all stages, the N28 transcript was more abundant than C22-4; moreover, the ratio of allele expression N28/C22-4 significantly increased during development (two-tailed *t*-test, *P* < 0.05. Table 1).

**Table 1:** Allele-specific expression assay

	First Leaf	Third Leaf	Fifth Leaf	Seventh Leaf
N28 expression proportion	0.52 (a)	0.57 (ab)	0.61 (b)	0.62 (b)

Results of allele specific expression assay, performed on the N28xC22-4 F1 hybrid line at four developmental stages. The values represent the proportion of the N28 allele transcript abundance over C22-4 transcript abundance. Different letters (a−b) indicate significant difference (*P* < 0.01, Fisher LSD). Letters shared between two groups (*i.e.*, a, ab) indicate a nonsignificant difference.

### Methylation at the CNS within *Vgt1* is conserved between maize and sorghum

**Figure 6:**
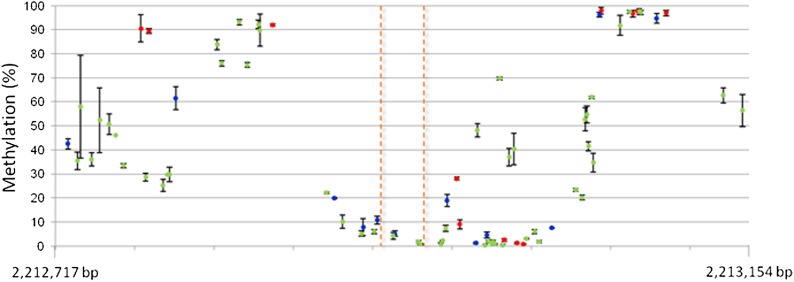
Results of the ultra-deep amplicon bisulfite sequencing of a region spanning the nucleotides 2,212,717 - 2,213,154 on sorghum chromosome 9 (JGI, v1.4) surrounding the CNS sequence in sorghum B.Tx623 at the V1 stage. On the y-axis, % of cytosine methylation as estimated by the Kismeth software. Methylation data points (mean values) are represented in different colors, according to cytosine context: red for CG, blue for CHG, green for CHH. The orange dotted lines highlight the CNS sequence. CNS, conserved noncoding sequence.

By means of bisulfite sequencing, we analyzed cytosine methylation at the sorghum 438-bp region including the CNS sequence which is syntenic to the maize *Vgt1*-associated CNS. DNA methylation was notably low at the four cytosines contained in the CNS itself (2.1%, on average) and at nearby cytosines, with values comparable with those detected at the CNS in N28 (1.1%). Moving away from the CNS, the level of methylation increased substantially on both sides (Figure 6 and Figure S9). Comparing the two stages of development, we observed that average methylation was higher in the V7 phase (*P* < 0.01).

## Discussion

### *Vgt1* pattern of methylation

Methylation analysis at *Vgt1* revealed that the N28 (late) and C22-4 (Gaspé Flint-originated, early) alleles differ significantly in cytosine methylation patterns. In particular, sequencing of the region encompassing the CNS/MITE sequence showed that the MITE transposon is heavily methylated and that this state is also present (at least at short distance) at cytosine residues lying outside the boundaries of the TE itself, including cytosines belonging to the CNS sequence in the early *Vgt1* allele. Furthermore, the methylation state of the two alleles is stably maintained, as the methylation pattern of single alleles did not change when assayed in F_1_ hybrid plants.

### MITE insertions can alter methylation at adjacent sites and impact gene expression

MITE transposon insertions such as the one observed at *Vgt1* have been repeatedly shown to be associated with QTL for agronomic traits in crops (Guillet-Claude *et al.* 2004; Magalhaes *et al.* 2007; Studer *et al.* 2011; Hou *et al.* 2012; Zerjal *et al.* 2012). In most cases, the MITE insertion seemed to act by disrupting a regulatory element of a nearby gene. The detailed molecular mechanisms by which a MITE insertion affects gene expression have not been elucidated and could include, besides the interruption of a transcription factor recognition sequence, the production of stable secondary chromatin structure (Bureau and Wessler 1992) and/or change in chromatin state due the action of siRNA, as shown by the fact that MITEs can be both target and source of siRNA (Kuang *et al.* 2009; Lu *et al.* 2011). Methylation changes induced by siRNA could also directly or indirectly affect the expression of nearby genes.

In plants, small interfering heterochromatic RNAs (24 nt in length) are involved in the formation of heterochromatin by guiding the action of the RNAi machinery against target DNA sequences (typically repeats and transposable elements), which as a result become methylated (Matzke *et al.* 2007; Daxinger *et al.* 2009; Hale *et al.* 2009; Simon and Meyers 2011). We looked for siRNA potentially targeting the *Vgt1* region using available small RNA libraries from the inbred line B73 (GEO accession number GSE17339) and the *mop1-1* mutant (GEO accession number GSE12173) (Nobuta *et al.* 2008); we aligned the reads using both *Vgt1* N28 and *Vgt1* C22-4 as reference sequences. A negligible number of hits were found when *Vgt1* N28 was the reference. As an example, with the B73 tassel library we observed that 1537 of the 1786 distinct sequences matching *Vgt1* C22-4 are 24-mers reads. In particular, the reads corresponding to the small RNA AAUCCACAUGGAUUGAGAGCUAAC, whose putative target sequence lies within the MITE transposon, represent over half of the sequences aligning against *Vgt1* C22-4. As expected, the abundance of such 24-mer is dramatically reduced in the *mop1-1* mutant. A siRNA directed against the same sequence was found to potentially target another MITE whose insertion at the *ZmV1-9* locus on chromosome 1 is associated to late male flowering phenotypes (Zerjal *et al.* 2012). This small RNA has hundreds of matches to many genomic locations, which precludes establishing an unambiguous connection to the *Vgt1* locus. This notwithstanding, it might play an important role in silencing and in accordance with our results we hypothesize that the binding of the small RNA is responsible for the heavy methylation of its target site within *Vgt1* and possibly of the methylation spreading to the adjacent sequences. As a supporting evidence to this hypothesis, our data showed that asymmetric CHH methylation, which is established and maintained via the RdDM pathway (Law and Jacobsen 2010), is extremely high across the MITE transposon.

Spreading of methylation from the MITE insertion to other regulatory sequences could be part of the molecular mechanism involved in translating the effect of MITE insertion in promoters and/or enhancers of *Vgt1*. In fact, heterochromatin spreading has been proposed as one of the mechanisms impacting gene regulation on a genome-wide scale as a consequence of transposon insertion in maize (Eichten *et al.* 2011).

Propagation of DNA methylation from transposons to adjacent regulatory sequences has been observed for at least another locus in maize, the *p1* gene, which is involved in the biosynthesis of phlobaphene pigments (Grotewold *et al.* 1994). In this case, the differential expression of the two epialleles *P1-rr* and *P1-pr* appears to arise from different cytosine methylation patterns at a distal enhancer located upstream the TSS, caused by a leakage of methylation from the hypermethylated nearby hAT and MULE elements that affects only the *P1-pr* silenced allele (Goettel and Messing 2013). Interestingly, also in this example spreading of methylation seemed to occur from transposons belonging to the MITE family, although this phenomenon did not contribute to obvious phenotypic variation.

### A complex network controls *ZmRap2.7* expression

The lack of a perfect correlation between levels of *ZmRap2.7* gene expression and genotype (and methylation state) at *Vgt1* is likely due to the complexity of *ZmRap2.7* gene expression regulation. Indeed, besides *Vgt1*, *ZmRap2.7* expression is likely to be under control of miR172 (given the presence of miR172 target sequence in *ZmRap2.7*), one of the most important and evolutionary ancient noncoding microRNAs (Park *et al.* 2002; Chen 2004), which seems to act by targeting mRNAs both by cleavage and translational repression (Zhu and Helliwell 2011). In our case, a different genotypic architecture for the miR172 family among B73 (five miR172 loci are present in the B73 genome. Zhu and Helliwell 2011), N28 and R22 could be responsible for differential effects on *ZmRap2.7* expression. Additionally, the expression of *ZmRap2.7* could be under feedback regulation as shown for the related gene *AP-2* in Arabidopsis (Schwab *et al.* 2005). Moreover, in addition to the CNS/MITE region, other sites within *Vgt1* might as well be involved in controlling *ZmRap2.7* expression: indeed, our results highlight at least one site, nucleotide C-1761, that shows not only differential methylation between the two alleles but also an increasing methylation during development in the N28 allele.

### Evolutionary conserved sequences share common features

*Vgt1-ZmRap2.7* was previously shown to correspond to a syntenic flowering time QTL in sorghum (short arm of chr. 9) with relatively high map resolution (1.9 cM, Mace *et al.* 2013; see also Brown *et al.* 2006 and Feltus *et al.* 2006). We previously found that a CNS was shared between maize *Vgt1* and the syntenic region in sorghum and rice (Salvi *et al.* 2007). Our methylation results now showed that the maize and sorghum *Vgt1*-related CNSs shared the same hypomethylation profile relative to surrounding regions, a distinguished feature of chromatin regions involved in the binding of transcription factors (Stadler *et al.* 2011; Thurman *et al.* 2012). Moreover, a survey of the DNA methylation maps of four rice cultivars (Li *et al.* 2012) revealed that a similar pattern of low methylation characterizes the CNS in this species as well (not shown).

### A model for *Vgt1* regulation of *ZmRap2.7* expression

Although our results do not provide conclusive evidence of a causal connection between differential methylation and *Vgt1* effect, they show a clear association between the MITE transposon insertion and hypermethylation at *Vgt1*. Based on these results, we propose a model in which, in the Gaspé-Flint allele, (i) the interruption of the CNS sequence by the insertion of the MITE transposon, (ii) the hypermethylation resulting from the presence of the MITE, or (iii) a combination of both these features can influence the interaction between *Vgt1* and *ZmRap2.7*. This alteration, in early stages of development prior to meristem transition, could reduce *ZmRap2.7* transcription, which eventually leads to early flowering. Further experimental evidence is needed to support the present hypothesis. One possibility is to investigate *Vgt1* function in an altered methylation context (*e.g.*, by the use of mutants in the methylation machinery, and/or by chemical modulators. Baubec *et al.* 2009; Law and Jacobsen 2010). Additionally, the study of DHS (DNaseI hypersensitivity) (Cockerill 2011) and histone modifications such as dimethylation of H3 lysine 9 or trimethylation of H3 lysine 27 (Locatelli *et al.* 2009) in the *Vgt1-ZmRap2.7* region would help to clarify if differential methylation observed at the two alleles is related to the level of chromatin compaction at *Vgt1* and how this state could be transmitted to the *ZmRap2.7* locus. The study of the three-dimensional architecture of the *Vgt1-ZmRap2.7* locus and long-range interactions between DNA sequences by chromosome conformation capture (Louwers *et al.* 2009; Crevillén *et al.* 2013) could also contribute to the understanding of the mechanism of action of *Vgt1*.

## 

## Supplementary Material

Supporting Information
