# Supplementary material for: A MITE Transposon Insertion Is Associated with Differential Methylation at the Maize Flowering Time QTL Vgt1
Source: G3 (Bethesda). 2014 Mar 7;4(5):805–12. doi: 10.1534/g3.114.010686 (PMC4025479; doi:10.1534/g3.114.010686)
Supplement: Supporting Information [file supp_4_5_805__index.html]

A MITE Transposon Insertion Is Associated with Differential Methylation at the Maize Flowering Time QTL Vgt1 — Supporting Information 

# A MITE Transposon Insertion Is Associated with Differential Methylation at the Maize Flowering Time QTL *Vgt1*

## Supporting Information for Castelletti *et al.*, 2014

**Files in this Data Supplement:**

- Supporting Information - Figures S1-S9, File S1, and Table S1 (PDF, 565 KB)
- Figure S1 - Schematic representation of the *Vgt1-ZmRap2.7* locus (Salvi et al., 2007) and of the PCR amplicons used for DNA methylation analysis. (PDF, 431 KB)
- Figure S2 - Density of methylation of the six amplicons within *Vgt1*, obtained based on McrBC/qPCR analysis. (PDF, 439 KB)
- Figure S3 - Average density of methylation of the six amplicons within *Vgt1*, obtained based on *Mcr*BC/qPCR analysis. (PDF, 314 KB)
- Figure S4 - Average levels of *Mcr*BC/qPCR based methylation (mean of six amplicons and four developmental stages) at *Vgt1* for the six maize lines utilized in this study. (PDF, 326 KB)
- Figure S5 - Average levels of *Mcr*BC/qPCR based methylation across the four developmental stages (mean of six maize lines and six amplicons). (PDF, 314 KB)
- Figure S6 - Interaction between amplicon methylation and developmental stage. (PDF, 336 KB)
- Figure S7 - Results of the ultra-deep amplicon bisulfite sequencing at the CNS/MITE region for the lines N28 and C22-4 for the stages V1, V3, V5 and V7. (PDF, 576 KB)
- Figure S8 - Results of the Sanger bisulfite sequencing at the CNS/MITE region within Vgt1 for the N28xC22-4 F1 hybrid line at the V1, V3, V5 and V7 stage. (PDF, 582 KB)
- Figure S9 - Results of the ultra-deep amplicon bisulfite sequencing of a region spanning the nucleotides 2,212,717-2,213,154 on sorghum chromosome 9 (JGI, v1.4) surrounding the CNS sequence in sorghum B.Tx623 at the V1 and V7 stage. (PDF, 346 KB)
- File S1 - *Mcr*BC methylation assay (Material and Methods and Results). (PDF, 169 KB)
- Table S1 - Primer list. (PDF, 114 KB)
