# Supplementary material for: A MITE Transposon Insertion Is Associated with Differential Methylation at the Maize Flowering Time QTL Vgt1
Source: G3 (Bethesda). 2014 Mar 7;4(5):805–12. doi: 10.1534/g3.114.010686 (PMC4025479; doi:10.1534/g3.114.010686)
Supplement: Supporting Information [file supp_g3.114.010686_010686SI.pdf]

G3·Genes | Genomes | Genetics

A MITE transposon insertion is associated with differential methylation at the maize flowering time QTL *Vgt1*

Authors: Sara Castelletti*,†, Roberto Tuberosa*, Massimo Pindo‡, Silvio Salvi*,§

Authors affiliations:
* Department of Agricultural Sciences (DipSA), University of Bologna, Bologna, Italy
† Present address : UMR de Génétique Végétale INRA – Univ Paris-Sud – CNRS, Gif-sur-Yvette, France
‡ Research and Innovation Centre, Foundation Edmund Mach, San Michele all'Adige, TN, Italy.

Corresponding author §:
Silvio Salvi
Address: Department of Agricultural Sciences (DipSA), University of Bologna, Viale Fanin 44, 40127 Bologna, Italy
Phone: +39-051-2096648
e-mail address: silvio.salvi@unibo.it

Author contributions:
S.C., S.S. and R.T. designed research; S.C. and M.P. performed research; S.C., S.S., M.P. and R.T. analyzed data; S.C. and S.S. wrote the manuscript.

DOI: 10.1534/g3.114.010686

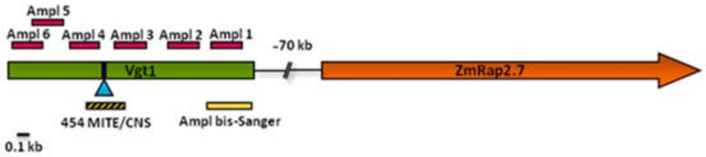

Figure S1 Schematic representation of the *Vgt1-ZmRap2.7* locus (Salvi et al., 2007) and of the PCR amplicons used for DNA methylation analysis. Red bars labelled from Ampl 1 to Ampl 6 indicate the genomic regions investigated for methylation level by McrBC/qPCR method. The yellow (Ampl bis-Sanger) and black/yellow (454 MITE/CNS) bars indicate regions investigated using the bisulfite sequencing approaches.

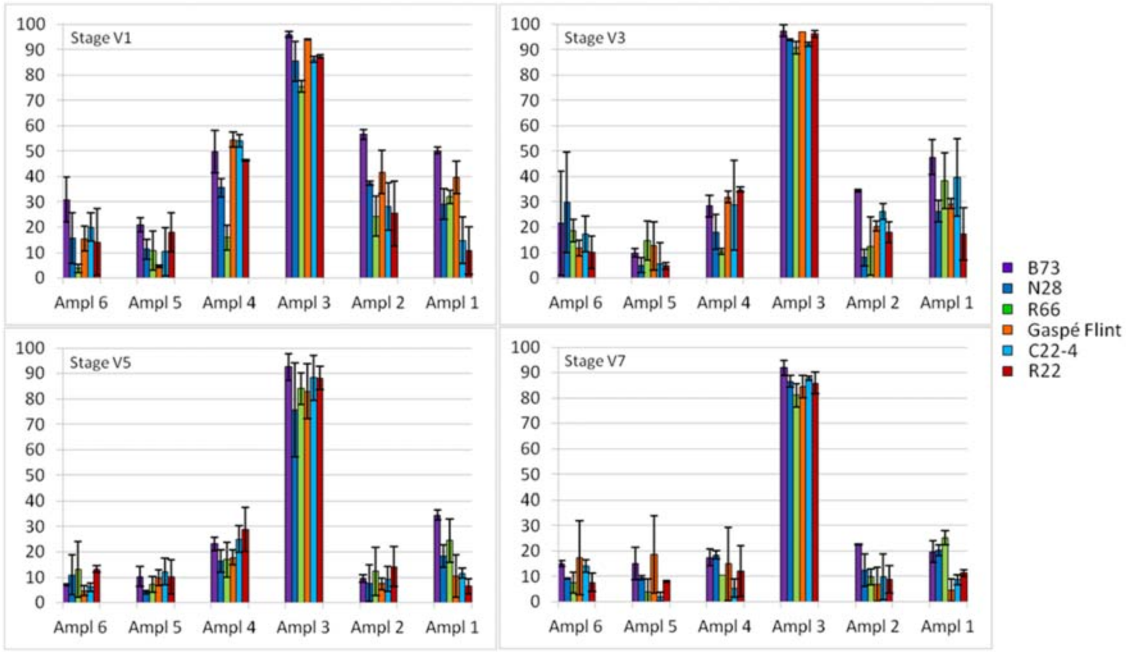

Figure S2 Density of methylation of the six amplicons within *Vgt1*, obtained based on McrBC/qPCR analysis. Mean values of density of methylation are represented as coloured bars; standard deviation values are shown as bars. Each stage of development (V1, V3, V5 and V7) is represented by a separate plot.

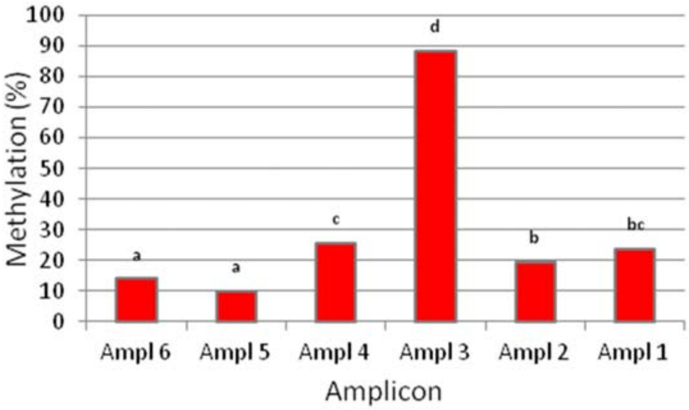

Figure S3 Average density of methylation of the six amplicons within *Vgt1*, obtained based on *Mcr*BC/qPCR analysis. Different letters (a, b) indicate significant differences (*P* <0.01, LSD). Letters shared between two groups (i.e. b, bc) indicate a non significant difference.

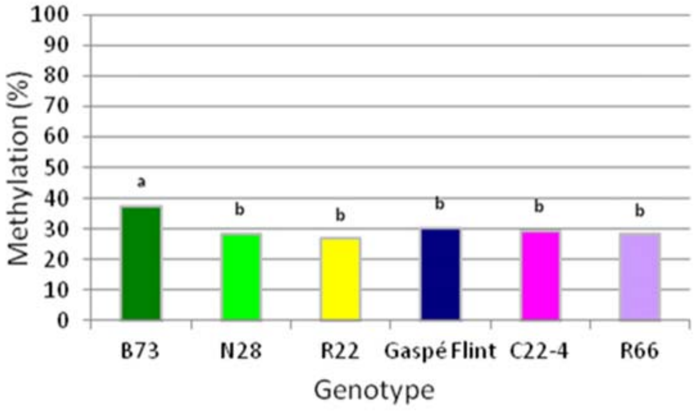

Figure S4 Average levels of *Mcr*BC/qPCR based methylation (mean of six amplicons and four developmental stages) at *Vgt1* for the six maize lines utilized in this study. Different letters (a, b) indicate significant differences (P <0.01, LSD).

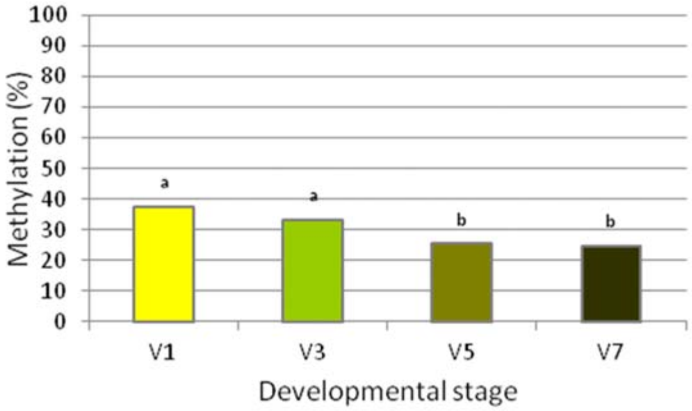

Figure S5 Average levels of *Mcr*BC/qPCR based methylation across the four developmental stages (mean of six maize lines and six amplicons). Different letters (a, b) indicate significant differences (*P* <0.01, LSD).

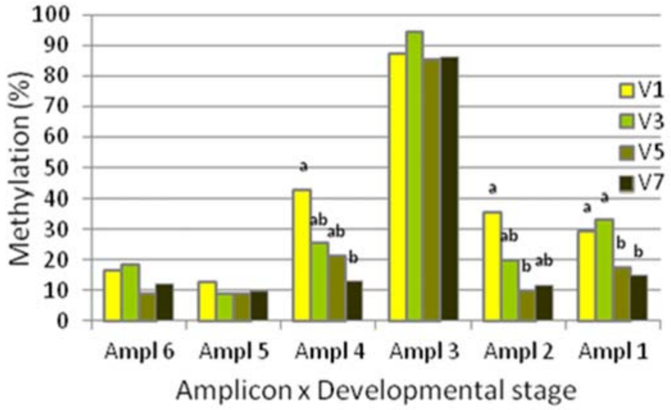

Figure S6 Interaction between amplicon methylation and developmental stage. Different letters (a, b) indicate significant differences (*P* <0.01, LSD). Letters shared between two groups (i.e. a, ab) indicate a non significant difference.

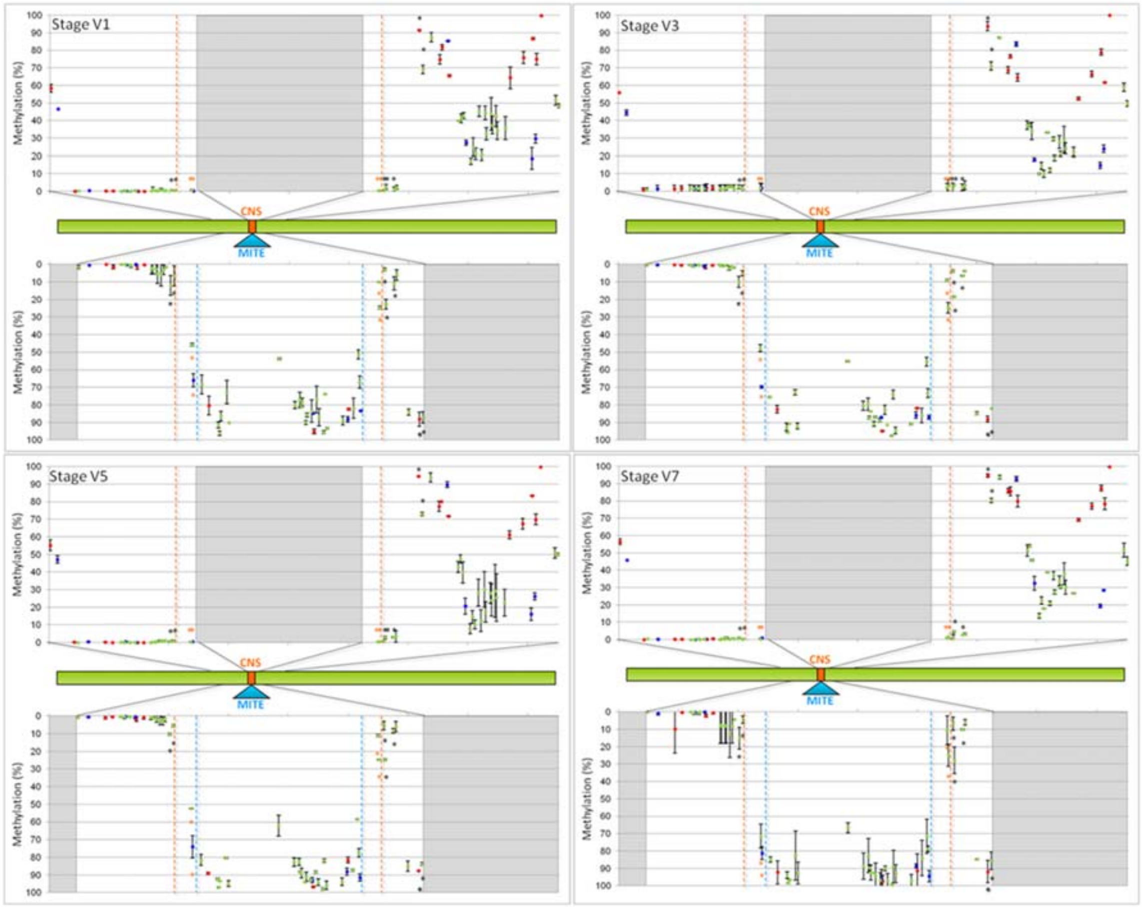

Figure S7 Results of the ultra-deep amplicon bisulfite sequencing at the CNS/MITE region for the lines N28 and C22-4 for the stages V1, V3, V5 and V7. Mean values are shown; standard deviation values are shown as bars. Methylation data points are represented in different colours, according to cytosine context: red for CG, blue for CHG, green for CHH. Top: methylation level (% of cytosine methylation as estimated by the Kismeth software, black vertical bars) for each cytosine within the sequence 617-920 bp of the N28 (late) allele. The gray block represents the site of MITE insertion (not present in the N28 allele). The orange dotted lines highlight the CNS sequence. Middle: the green bar represents the N28-Vgt1 locus, with black dotted lines indicating the regions for which methylation has been explored in this experiment.Bottom: methylation level estimated for each cytosine of the C22-4 (early) allele within the region corresponding to the sequence 643-792 bp of the N28 allele. The gray blocks define regions within Vgt1 that have not been tested in this analysis for the C22-4 allele with respect to N28. The light blue dotted lines delimitate the MITE insertion, which is present in C22-4 only. The black * indicates a significantly differentially methylated cytosine between N28 and C22-4 (P <0.01, LSD). The red * indicates significant difference in methylation at the cytosine included in the CNS region.

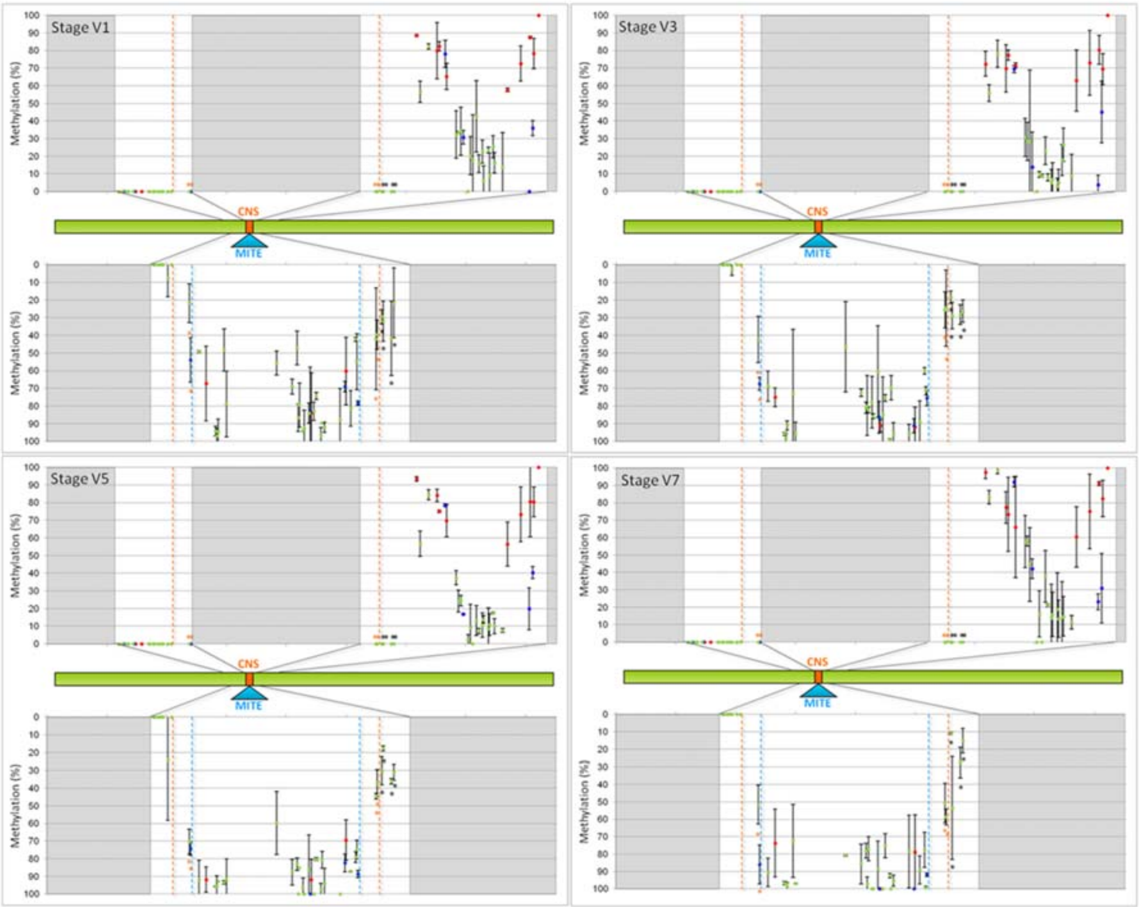

Figure S8 Results of the Sanger bisulfite sequencing at the CNS/MITE region within Vgt1 for the N28xC22-4 F1 hybrid line at the V1, V3, V5 and V7 stage. Mean values are shown; standard deviation values are shown as bars. Methylation data points are represented in different colours, according to cytosine context: red for CG, blue for CHG, green for CHH. Top: methylation level (% of cytosine methylation as estimated by the Mutation Surveyor software, black vertical bars) estimated for each cytosine of the N28 (late) allele. Middle: the green bar represents the N28-Vgt1 locus, with black dotted lines indicating the regions for which methylation has been explored in this experiment. Bottom: methylation level estimated for each cytosine of the C22-4 (early) allele. The two light blue dotted lines indicate the MITE insertion, the orange dotted lines highlight the CNS sequence. The black * indicates a significantly differentially methylated cytosine between N28 and C22-4 (P <0.01, two tailed t-test). The red * indicates a significant difference in methylation at the cytosine included in the CNS region.

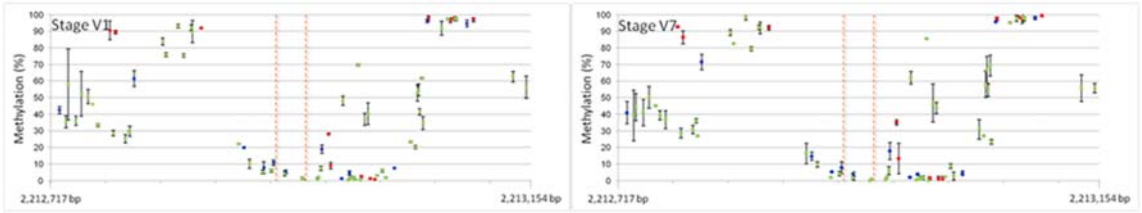

Figure S9 Results of the ultra-deep amplicon bisulfite sequencing of a region spanning the nucleotides 2,212,717-2,213,154 on sorghum chromosome 9 (JGI, v1.4) surrounding the CNS sequence in sorghum B.Tx623 at the V1 and V7 stage. On the y-axis, % of cytosine methylation as estimated by the Kismeth software. Methylation data points (mean values) are represented in different colours, according to cytosine context: red for CG, blue for CHG, green for CHH. The orange dotted lines highlight the CNS sequence.

File S1

***Mcr*BC methylation assay**

Material and methods

Genomic DNA was extracted following a standard CTAB method as described in (Saghai-Maroof *et al.* 1984); each DNA sample was diluted in double-distilled water to a final concentration of 20 ng/µl and fragmented using a Digital Sonifier S-450D (Branson, Danbury, CT, USA) with the following parameters: amplitude 20%, 10 sec for four times. For each sample, 1 µg of DNA was digested overnight at 37° with 50 units of *Mcr*BC (New England Biolabs, Beverly, MA, USA) or 50% glycerol (mock samples), 1X NEB 2 buffer, 1X bovine serum albumin, 1X GTP in a final volume of 60 µl; the enzyme was deactivated at 65° for 20 min. PCR primer pairs were designed with the Primer3 software (http://frodo.wi.mit.edu/) using default settings (Fig. S1 and Tab. S1). Digested and mock samples were diluted 1:8 (v/v) and then used as template in Real-Time PCR reactions with Platinum® SYBR® Green qPCR SuperMix-UDG (Invitrogen, Carlsbad, CA, USA) and with the following conditions: 40 cycles at 95° for 10'' and 60° for 1'. *Mcr*BC digests genomic DNA in presence of at least two methylated cytosine residues in the context 5′-Pu^{m}C (N40–3000) Pu^{m}C-3′; the extent of DNA digestion is proportional to the level of cytosine methylation, which allows for the estimation of the density of DNA methylation for each investigated genomic fragment. Density of cytosine methylation for each sample was then calculated on the basis of the ΔCT between mock and digested DNA (Telias *et al.* 2011), using the formula

$$\text{Percentage of methylation} = 100 - \frac{100}{\text{Efficiency}^{-\Delta CT}}$$

ANOVA and Fisher's LSD were applied to compare methylation levels among stages, amplicons and genotypes.

Results

To investigate the dynamics of *Vgt1* DNA methylation levels, we first utilized a method based on restriction with the methylation-dependent enzyme *Mcr*BC followed by qPCR. The analyses were carried out on the same samples utilized for gene expression, and on six targeted regions (average size 243 bp) spanning the *Vgt1* locus (Fig. S1). Ampl 3 region (838-1089 bp) showed a much higher methylation than all other regions at all stages and in all genotypes (P <0.01. Fig. S2). The regions corresponding to Ampl 4 (486-721 bp) and Ampl 2 (1262-1505 bp) were more methylated compared to Ampl 5 (187-438 bp) and Ampl 6 (34-270 bp) (Fig. S1). A significantly (P <0.01) higher methylation across amplicons was recorded for B73 as compared to all other lines (Fig. S4). For all the genotypes, a decrease in the levels of methylation was detected along the transition from younger to older tissues (first leaf and third leaf > fifth leaf and seventh leaf; P <0.01) (Fig. S5). Additionally, the methylation levels of Ampl 1, 2 and 4 regions underwent a substantial reduction from the younger stages (first and third leaf) to the older ones (fifth and seventh leaf) whereas the methylation level of Ampl 3, 5 and 6 remained

nearly unchanged over time (*P* <0.01) (Fig. S6). The CNS (743-761 bp in the N28 allele of *Vgt1*) and the MITE insertion (in the C22-4 allele) lie between Ampl 3 (838-1089 bp) and Ampl 4 (486-721 bp) but were not considered in this analysis as it was not possible to design suitable primer pairs due to technical constraints. However, as *Mcr*BC cutting site is highly variable in terms of distance between two methylated Cs, it is possible that the density of methylation of the CNS/MITE region might have influenced the signal revealed for the two flanking amplicons. So, although with this approach the late and early *Vgt1* alleles did not show a significant difference in methylation level, the high and constant methylation levels observed for Ampl 3 seemed to point out the CNS/MITE region and the nearby sequences as a preferred target for methylation.

Table S1 Primer list

Primer/probe name	Sequence
ZmRap2.7_RT_for	CTTCTTCGTCTTCACAAACCA
ZmRap2.7_RT_rev	CTTCCCGGCAGATTTACAGT
*aat*_F	ATGGGGTATGGCGAGGAT
*aat*_R	TTGCACGACGAGCTAAAGACT
Ampl1_for	AGATCATCAGTTCCAGTTCGAGA
Ampl1_rev	TCTGCCTCAGCTAGAAAAATCG
Ampl2_for	CGTCAAATCCATCATCGTCA
Ampl2_rev	GTCACGAGGTTAAATACAGCTTCC
Ampl3_for	ATGTTGGAGCAAGAAGAAGCA
Ampl3_rev	ACTTCACATCCATTCCATCCA
Ampl4_for	ATGTGTGAAGGTAGGCAAACG
Ampl4_rev	GCCGTCTCAAGGGACAAGT
Ampl5_for	GCCGTGTCCAACAGGAAG
Ampl5_rev	GCACTGGCACTGCACTTG
Ampl6_for	GACGGCCTCTGCTACTGCTA
Ampl6_rev	CGCGCGTTCCTTTCTTTAT
Ampl Bis-Sanger _N28_for	AAGTGGAYTYGATGGATGGGAATG
Ampl Bis-Sanger _N28_rev	AAAATAATARTRTTTACCTTCAACC
Ampl Bis-Sanger _C224_for	GATTTGAYGTTAATTGYTTYTTTGT
Ampl Bis-Sanger _C224_rev	AAARCTARTCTATTTARATCATCA
CNS_for	GAAAAAGYGGGTGYAGGTATGAAAG
CNS_rev	ATAAATCTATRTARATCACTCCTACGA
MITE_for	GTYAAAAGGAGYAGGAGAGGAGA
MITE_rev	TRTCRCTTTCCATRAATAAACA
CNS_Sorghum_for	TGGGTTAYTGTAGYAYTTAAGG
CNS_Sorghum_rev	CCAATRATACCAATTATRTACTATA
ZmRap2.7_F	TCGACGATGCTCCCTCTGA
ZmRap2.7_R	GGCCGGCGGATGCT
ZmRap2.7_VIC	CCTCGTCGGCTGTC
ZmRap2.7_FAM	CCTCGTCAGCTGTC

List of primer/probe names and relative sequences
