# Supplementary material for: A MITE Transposon Insertion Is Associated with Differential Methylation at the Maize Flowering Time QTL Vgt1
Source: G3 (Bethesda). 2014 Mar 7;4(5):805–12. doi: 10.1534/g3.114.010686 (PMC4025479; doi:10.1534/g3.114.010686)
Supplement: Supporting Information [file supp_g3.114.010686_FigureS1.pdf]

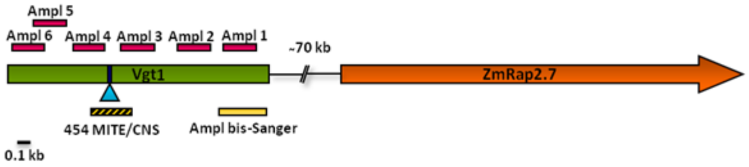

Figure S1 Schematic representation of the *Vgt1-ZmRap2.7* locus (Salvi et al., 2007) and of the PCR amplicons used for DNA methylation analysis. Red bars labelled from Ampl 1 to Ampl 6 indicate the genomic regions investigated for methylation level by McrBC/qPCR method. The yellow (Ampl bis-Sanger) and black/yellow (454 MITE/CNS) bars indicate regions investigated using the bisulfite sequencing approaches.
