# Supplementary material for: A MITE Transposon Insertion Is Associated with Differential Methylation at the Maize Flowering Time QTL Vgt1
Source: G3 (Bethesda). 2014 Mar 7;4(5):805–12. doi: 10.1534/g3.114.010686 (PMC4025479; doi:10.1534/g3.114.010686)
Supplement: Supporting Information [file supp_g3.114.010686_FigureS2.pdf]

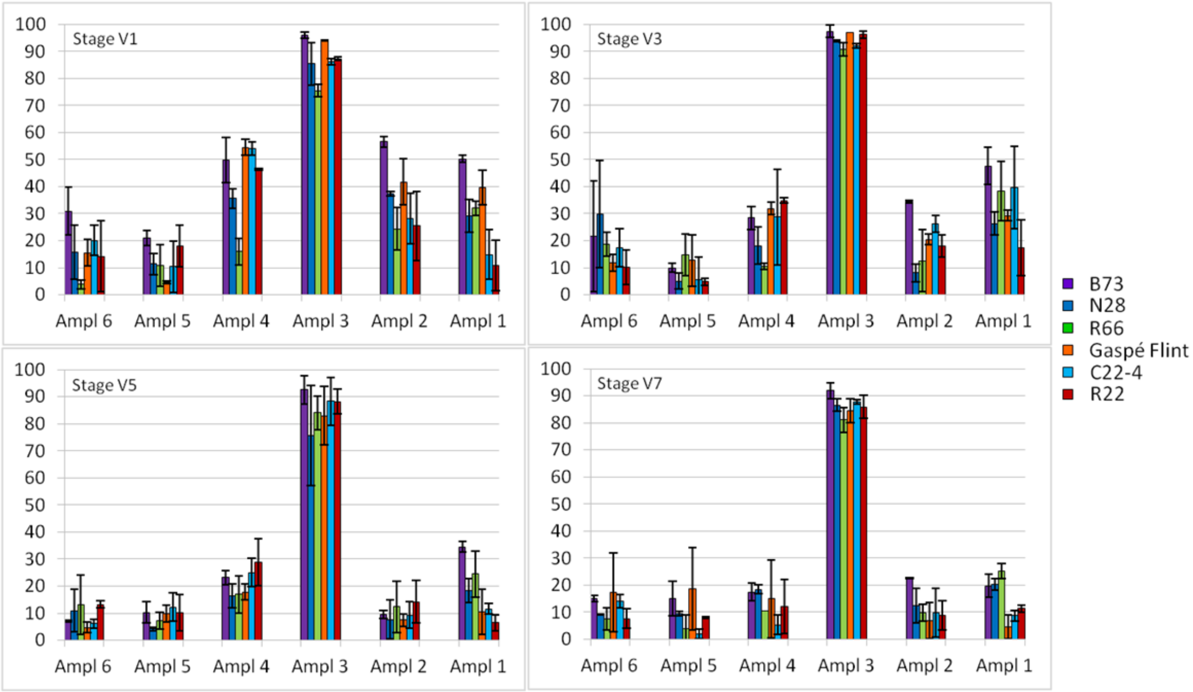

Figure S2 Density of methylation of the six amplicons within *Vgt1*, obtained based on McrBC/qPCR analysis. Mean values of density of methylation are represented as coloured bars; standard deviation values are shown as bars. Each stage of development (V1, V3, V5 and V7) is represented by a separate plot.
