# Supplementary material for: A MITE Transposon Insertion Is Associated with Differential Methylation at the Maize Flowering Time QTL Vgt1
Source: G3 (Bethesda). 2014 Mar 7;4(5):805–12. doi: 10.1534/g3.114.010686 (PMC4025479; doi:10.1534/g3.114.010686)
Supplement: Supporting Information [file supp_g3.114.010686_FigureS5.pdf]

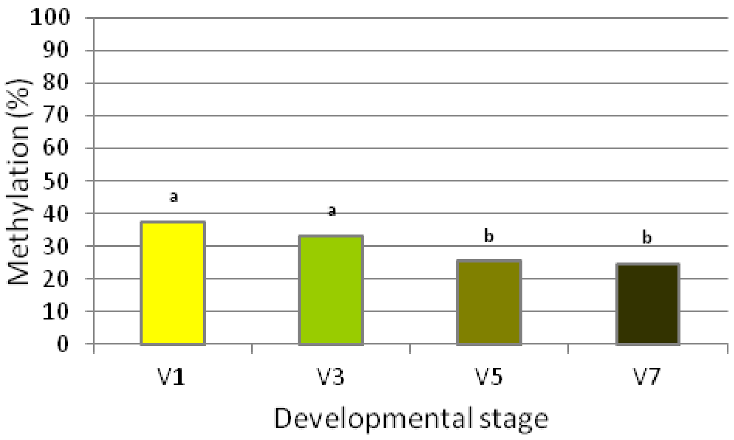

Figure S5 Average levels of *Mcr*BC/qPCR based methylation across the four developmental stages (mean of six maize lines and six amplicons). Different letters (a, b) indicate significant differences (P <0.01, LSD).
