# Supplementary material for: A MITE Transposon Insertion Is Associated with Differential Methylation at the Maize Flowering Time QTL Vgt1
Source: G3 (Bethesda). 2014 Mar 7;4(5):805–12. doi: 10.1534/g3.114.010686 (PMC4025479; doi:10.1534/g3.114.010686)
Supplement: Supporting Information [file supp_g3.114.010686_FigureS6.pdf]

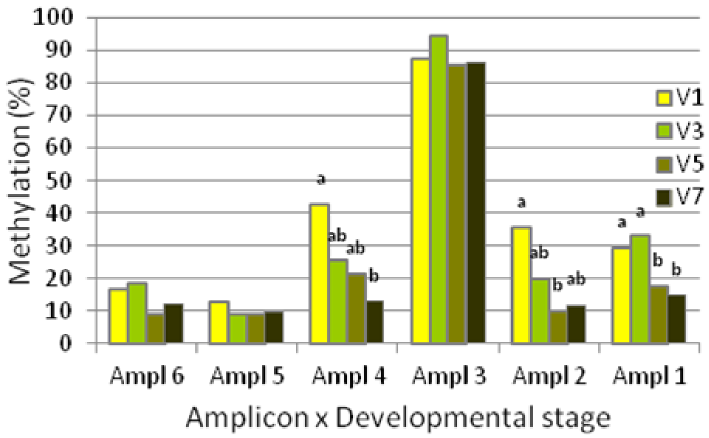

Figure S6 Interaction between amplicon methylation and developmental stage. Different letters (a, b) indicate significant differences (*P* <0.01, LSD). Letters shared between two groups (i.e. a, ab) indicate a non significant difference.
