# Supplementary material for: A MITE Transposon Insertion Is Associated with Differential Methylation at the Maize Flowering Time QTL Vgt1
Source: G3 (Bethesda). 2014 Mar 7;4(5):805–12. doi: 10.1534/g3.114.010686 (PMC4025479; doi:10.1534/g3.114.010686)
Supplement: Supporting Information [file supp_g3.114.010686_FigureS7.pdf]

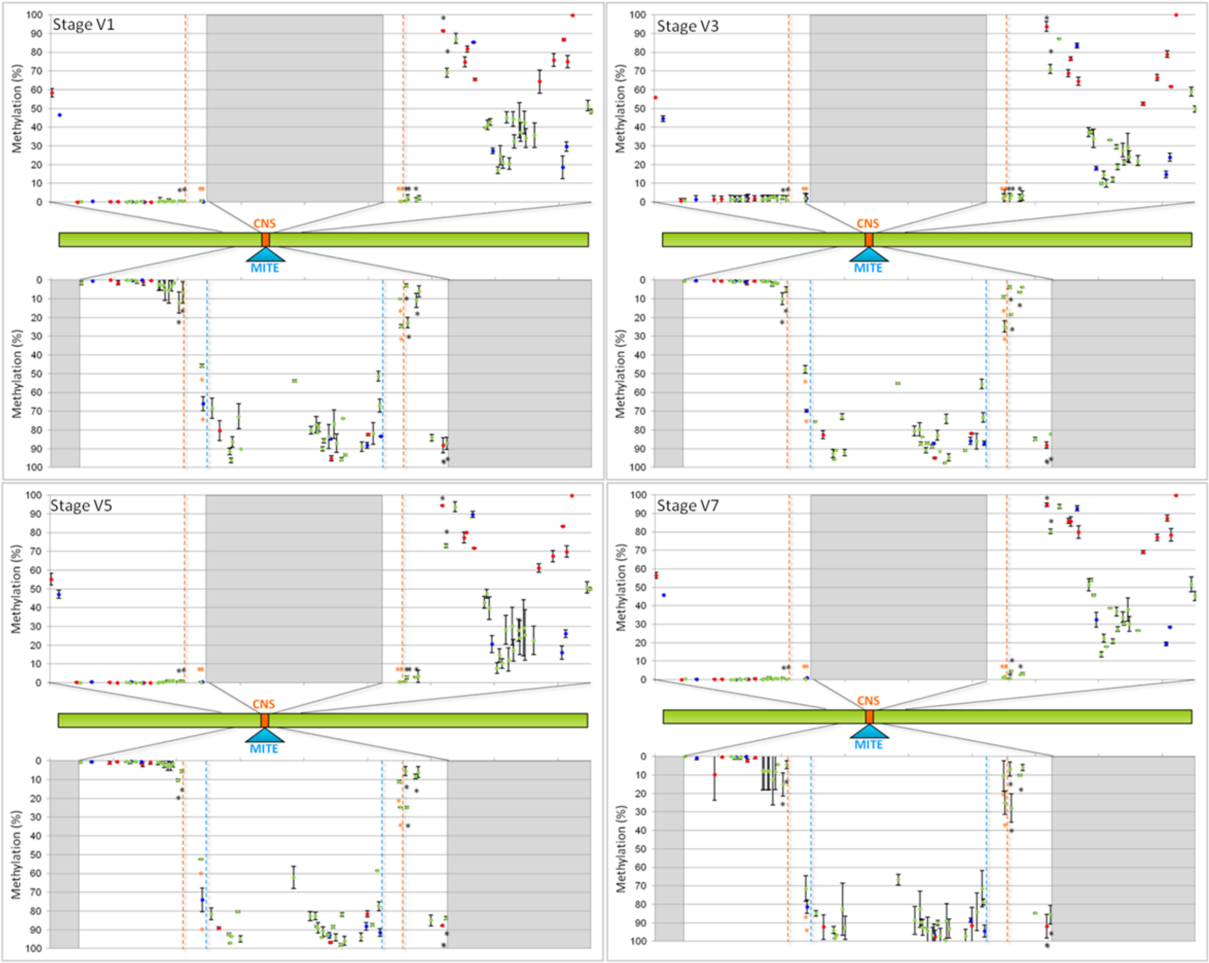

Figure S7 Results of the ultra-deep amplicon bisulfite sequencing at the CNS/MITE region for the lines N28 and C22-4 for the stages V1, V3, V5 and V7. Mean values are shown; standard deviation values are shown as bars. Methylation data points are represented in different colours, according to cytosine context: red for CG, blue for CHG, green for CHH. Top: methylation level (% of cytosine methylation as estimated by the Kismeth software, black vertical bars) for each cytosine within the sequence 617-920 bp of the N28 (late) allele. The gray block represents the site of MITE insertion (not present in the N28 allele). The orange dotted lines highlight the CNS sequence. Middle: the green bar represents the N28-Vgt1 locus, with black dotted lines indicating the regions for which methylation has been explored in this experiment.Bottom: methylation level estimated for each cytosine of the C22-4 (early) allele within the region corresponding to the sequence 643-792 bp of the N28 allele. The gray blocks define regions within Vgt1 that have not been tested in this analysis for the C22-4 allele with respect to N28. The light blue dotted lines delimitate the MITE insertion, which is present in C22-4 only. The black * indicates a significantly differentially methylated cytosine between N28 and C22-4 (P <0.01, LSD). The red * indicates significant difference in methylation at the cytosine included in the CNS region.
