# Supplementary material for: A MITE Transposon Insertion Is Associated with Differential Methylation at the Maize Flowering Time QTL Vgt1
Source: G3 (Bethesda). 2014 Mar 7;4(5):805–12. doi: 10.1534/g3.114.010686 (PMC4025479; doi:10.1534/g3.114.010686)
Supplement: Supporting Information [file supp_g3.114.010686_FigureS8.pdf]

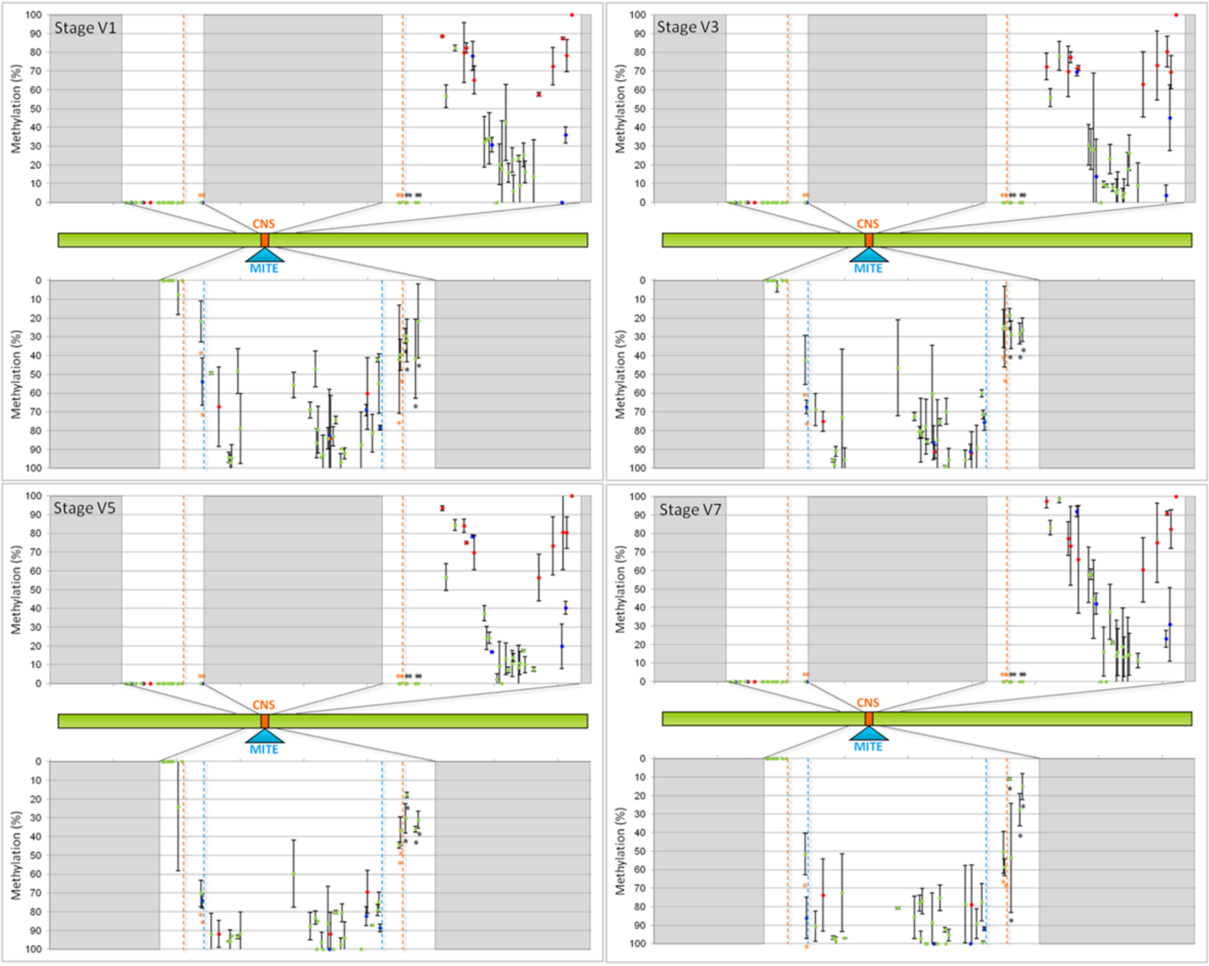

Figure S8 Results of the Sanger bisulfite sequencing at the CNS/MITE region within Vgt1 for the N28xC22-4 F1 hybrid line at the V1, V3, V5 and V7 stage. Mean values are shown; standard deviation values are shown as bars. Methylation data points are represented in different colours, according to cytosine context: red for CG, blue for CHG, green for CHH. Top: methylation level (% of cytosine methylation as estimated by the Mutation Surveyor software, black vertical bars) estimated for each cytosine of the N28 (late) allele. Middle: the green bar represents the N28-Vgt1 locus, with black dotted lines indicating the regions for which methylation has been explored in this experiment. Bottom: methylation level estimated for each cytosine of the C22-4 (early) allele. The two light blue dotted lines indicate the MITE insertion, the orange dotted lines highlight the CNS sequence. The black * indicates a significantly differentially methylated cytosine between N28 and C22-4 (P <0.01, two tailed t-test). The red * indicates a significant difference in methylation at the cytosine included in the CNS region.
