# Supplementary material for: A MITE Transposon Insertion Is Associated with Differential Methylation at the Maize Flowering Time QTL Vgt1
Source: G3 (Bethesda). 2014 Mar 7;4(5):805–12. doi: 10.1534/g3.114.010686 (PMC4025479; doi:10.1534/g3.114.010686)
Supplement: Supporting Information [file supp_g3.114.010686_FigureS9.pdf]

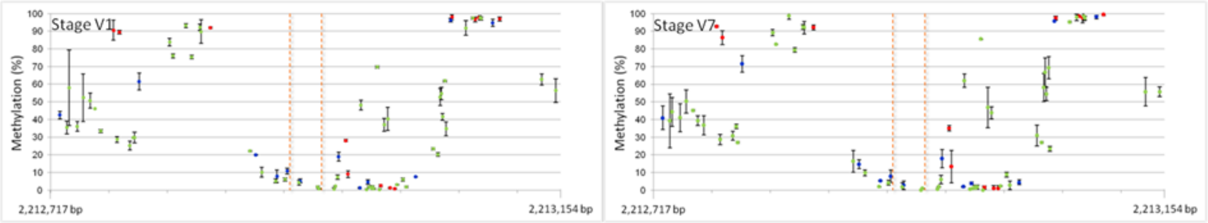

Figure S9 Results of the ultra-deep amplicon bisulfite sequencing of a region spanning the nucleotides 2,212,717-2,213,154 on sorghum chromosome 9 (JGI, v1.4) surrounding the CNS sequence in sorghum B.Tx623 at the V1 and V7 stage. On the y-axis, % of cytosine methylation as estimated by the Kismeth software. Methylation data points (mean values) are represented in different colours, according to cytosine context: red for CG, blue for CHG, green for CHH. The orange dotted lines highlight the CNS sequence.
