# Supplementary material for: A MITE Transposon Insertion Is Associated with Differential Methylation at the Maize Flowering Time QTL Vgt1
Source: G3 (Bethesda). 2014 Mar 7;4(5):805–12. doi: 10.1534/g3.114.010686 (PMC4025479; doi:10.1534/g3.114.010686)
Supplement: Supporting Information [file supp_g3.114.010686_FileS1.pdf]

File S1

***Mcr*BC methylation assay**

Material and methods

Genomic DNA was extracted following a standard CTAB method as described in (Saghai-Maroof *et al.* 1984); each DNA sample was diluted in double-distilled water to a final concentration of 20 ng/µl and fragmented using a Digital Sonifier S-450D (Branson, Danbury, CT, USA) with the following parameters: amplitude 20%, 10 sec for four times. For each sample, 1 µg of DNA was digested overnight at 37° with 50 units of *Mcr*BC (New England Biolabs, Beverly, MA, USA) or 50% glycerol (mock samples), 1X NEB 2 buffer, 1X bovine serum albumin, 1X GTP in a final volume of 60 µl; the enzyme was deactivated at 65° for 20 min. PCR primer pairs were designed with the Primer3 software (http://frodo.wi.mit.edu/) using default settings (Fig. S1 and Tab. S1). Digested and mock samples were diluted 1:8 (v/v) and then used as template in Real-Time PCR reactions with Platinum® SYBR® Green qPCR SuperMix-UDG (Invitrogen, Carlsbad, CA, USA) and with the following conditions: 40 cycles at 95° for 10'' and 60° for 1'. *Mcr*BC digests genomic DNA in presence of at least two methylated cytosine residues in the context 5′-Pu^{m}C (N40–3000) Pu^{m}C-3′; the extent of DNA digestion is proportional to the level of cytosine methylation, which allows for the estimation of the density of DNA methylation for each investigated genomic fragment. Density of cytosine methylation for each sample was then calculated on the basis of the ΔCT between mock and digested DNA (Telias *et al.* 2011), using the formula

$$\text{Percentage of methylation} = 100 - \frac{100}{\text{Efficiency}^{-\Delta CT}}$$

ANOVA and Fisher's LSD were applied to compare methylation levels among stages, amplicons and genotypes.

Results

To investigate the dynamics of *Vgt1* DNA methylation levels, we first utilized a method based on restriction with the methylation-dependent enzyme *Mcr*BC followed by qPCR. The analyses were carried out on the same samples utilized for gene expression, and on six targeted regions (average size 243 bp) spanning the *Vgt1* locus (Fig. S1). Ampl 3 region (838-1089 bp) showed a much higher methylation than all other regions at all stages and in all genotypes (P <0.01. Fig. S2). The regions corresponding to Ampl 4 (486-721 bp) and Ampl 2 (1262-1505 bp) were more methylated compared to Ampl 5 (187-438 bp) and Ampl 6 (34-270 bp) (Fig. S1). A significantly (P <0.01) higher methylation across amplicons was recorded for B73 as compared to all other lines (Fig. S4). For all the genotypes, a decrease in the levels of methylation was detected along the transition from younger to older tissues (first leaf and third leaf > fifth leaf and seventh leaf; P <0.01) (Fig. S5). Additionally, the methylation levels of Ampl 1, 2 and 4 regions underwent a substantial reduction from the younger stages (first and third leaf) to the older ones (fifth and seventh leaf) whereas the methylation level of Ampl 3, 5 and 6 remained

nearly unchanged over time (*P* <0.01) (Fig. S6). The CNS (743-761 bp in the N28 allele of *Vgt1*) and the MITE insertion (in the C22-4 allele) lie between Ampl 3 (838-1089 bp) and Ampl 4 (486-721 bp) but were not considered in this analysis as it was not possible to design suitable primer pairs due to technical constraints. However, as *Mcr*BC cutting site is highly variable in terms of distance between two methylated Cs, it is possible that the density of methylation of the CNS/MITE region might have influenced the signal revealed for the two flanking amplicons. So, although with this approach the late and early *Vgt1* alleles did not show a significant difference in methylation level, the high and constant methylation levels observed for Ampl 3 seemed to point out the CNS/MITE region and the nearby sequences as a preferred target for methylation.
