# Supplementary material for: A MITE Transposon Insertion Is Associated with Differential Methylation at the Maize Flowering Time QTL Vgt1
Source: G3 (Bethesda). 2014 Mar 7;4(5):805–12. doi: 10.1534/g3.114.010686 (PMC4025479; doi:10.1534/g3.114.010686)
Supplement: Supporting Information [file supp_g3.114.010686_TableS1.pdf]

Table S1 Primer list

Primer/probe name	Sequence
ZmRap2.7_RT_for	CTTCTTCGTCTTCACAAACCA
ZmRap2.7_RT_rev	CTTCCCGGCAGATTTACAGT
*aat*_F	ATGGGGTATGGCGAGGAT
*aat*_R	TTGCACGACGAGCTAAAGACT
Ampl1_for	AGATCATCAGTTCCAGTTCGAGA
Ampl1_rev	TCTGCCTCAGCTAGAAAAATCG
Ampl2_for	CGTCAAATCCATCATCGTCA
Ampl2_rev	GTCACGAGGTTAAATACAGCTTCC
Ampl3_for	ATGTTGGAGCAAGAAGAAGCA
Ampl3_rev	ACTTCACATCCATTCCATCCA
Ampl4_for	ATGTGTGAAGGTAGGCAAACG
Ampl4_rev	GCCGTCTCAAGGGACAAGT
Ampl5_for	GCCGTGTCCAACAGGAAG
Ampl5_rev	GCACTGGCACTGCACTTG
Ampl6_for	GACGGCCTCTGCTACTGCTA
Ampl6_rev	CGCGCGTTCCTTTCTTTAT
Ampl Bis-Sanger _N28_for	AAGTGGAYTYGATGGATGGGAATG
Ampl Bis-Sanger _N28_rev	AAAATAATARTRTTTACCTTCAACC
Ampl Bis-Sanger _C224_for	GATTTGAYGTTAATTGYTTYTTTGT
Ampl Bis-Sanger _C224_rev	AAARCTARTCTATTTARATCATCA
CNS_for	GAAAAAGYGGGTGYAGGTATGAAAG
CNS_rev	ATAAATCTATRTARATCACTCCTACGA
MITE_for	GTYAAAAGGAGYAGGAGAGGAGA
MITE_rev	TRTCRCTTTCCATRAATAAACA
CNS_Sorghum_for	TGGGTTAYTGTAGYAYTTAAGG
CNS_Sorghum_rev	CCAATRATACCAATTATRTACTATA
ZmRap2.7_F	TCGACGATGCTCCCTCTGA
ZmRap2.7_R	GGCCGGCGGATGCT
ZmRap2.7_VIC	CCTCGTCGGCTGTC
ZmRap2.7_FAM	CCTCGTCAGCTGTC

List of primer/probe names and relative sequences
